# Penetrating Neck Injury: A Case Report and Review of Management

**DOI:** 10.7759/cureus.92868

**Published:** 2025-09-21

**Authors:** Chandu Raj B, Madhur Uniyal, Arun Varghese, Nilesh Y Jagne, Deepika Kandpal

**Affiliations:** 1 Nursing, All India Institute of Medical Sciences, Rishikesh, Rishikesh, IND; 2 Trauma Surgery and Critical Care, All India Institute of Medical Sciences, Rishikesh, Rishikesh, IND; 3 College of Nursing, All India Institute of Medical Sciences, Gorakhpur, Gorakhpur, IND; 4 Trauma and Emergency, All India Institute of Medical Sciences, Nagpur, Nagpur, IND

**Keywords:** advanced trauma life support, airway management, cricoid membrane tear, emergency trauma care, laryngotracheal injury, pediatric trauma, penetrating neck injury, primary survey, tracheal injury, zones of neck injury

## Abstract

Penetrating neck injuries (PNIs), although infrequent, are critical emergencies due to the intricate anatomy and presence of vital structures within the neck. PNIs are often caused by sharp objects or projectiles, posing a high risk of airway compromise or major vascular damage, leading to significant morbidity and mortality. Despite the severity of PNIs, international protocols for optimal management remain ambiguous, with most approaches still relying on zonal classification techniques. Traditional management strategies have shifted from mandatory surgical exploration to a more selective approach guided by clinical assessment and advanced imaging. Here, we report a case of a four-year-old girl with a PNI involving a cricoid membrane tear. The patient was successfully managed with selective surgical intervention and airway stabilisation via video laryngoscopic intubation. This case emphasises the challenges associated with PNIs in pediatric patients, highlighting the importance of individualised, timely interventions. The ambiguity in standardised guidelines underscores the need for comprehensive assessment and tailored treatment strategies. Our case also underscores the critical importance of rapid airway management, multidisciplinary collaboration, and preventive measures to mitigate risks, especially in young children.

## Introduction

Penetrating neck injuries (PNIs) are rare, but they create life-threatening emergencies that need quick identification and immediate intervention [1]. The neck contains numerous vital structures, and managing these injuries can be challenging and complex. However, ambiguity exists in international consensus standards for managing PNIs, and most approaches still rely on traditional zonal techniques [2]. These injuries often result from sharp objects like knives, scissors, glass, or gunshots, making up to 5-10% of emergency trauma cases [3]. PNIs have a mortality rate of up to 10%, which can be aggravated by severe bleeding from damaged blood vessels [3,4]. If a wound track crosses the trachea, it might cause tracheal damage. About 25% of these injuries cause significant vascular damage, leading to a high death rate of up to 50% [4]. Injuries to the tracheobronchial tree happen in 10-20% of cases, with a 20% death rate [5]. These cases can lead to conditions like pneumomediastinum, subcutaneous emphysema, and trapped air near the trachea. Most of these injuries occur in a specific neck area known as zone II, which runs from the cricoid cartilage to the mandibular angle in adults and children [6]. These injuries are complex and can involve damage to blood vessels, block airways, injure the oesophagus, and cause severe sympathetic stimulation.

The first steps in managing patients with these injuries focus on maintaining their airway and circulation, following the guidelines of the Advanced Trauma Life Support (ATLS) protocol [7]. The ATLS protocol provides a systematic approach to managing trauma patients, beginning with airway management and cervical spine protection, followed by assessment of breathing, circulation with haemorrhage control, and exposure with temperature regulation. The protocol emphasises that airway obstruction poses the most immediate threat to life and must be addressed before breathing or circulatory issues. In the present case, early airway intervention and structured resuscitation, following ATLS principles, played a critical role in optimising patient outcomes. Treating these injuries within the first hour, often called the golden hour, can prevent complications like aspiration and improve outcomes [7]. The approach to treating PNIs has shifted from mandatory exploration to more selective management [6]. The medical team usually prefers carefully considering surgery based on physical examination and specific tests. If the airway is not stable, immediate actions like endotracheal intubation or creating an emergent surgical airway, such as tracheostomy, might be necessary. Care must be taken, especially with children, to avoid damage to the cricoid cartilage, which is the only circumferential support for the upper trachea. For this reason, surgical cricothyroidotomy is not recommended for children under 12 years of age [8].

While prompt and effective management is crucial, the optimal approach to these injuries remains debated. In this context, we present a case of a PNI involving cricoid membrane damage. This case highlights the unique challenges in management, particularly in resource-limited settings.

## Case presentation

**Figure 1:**
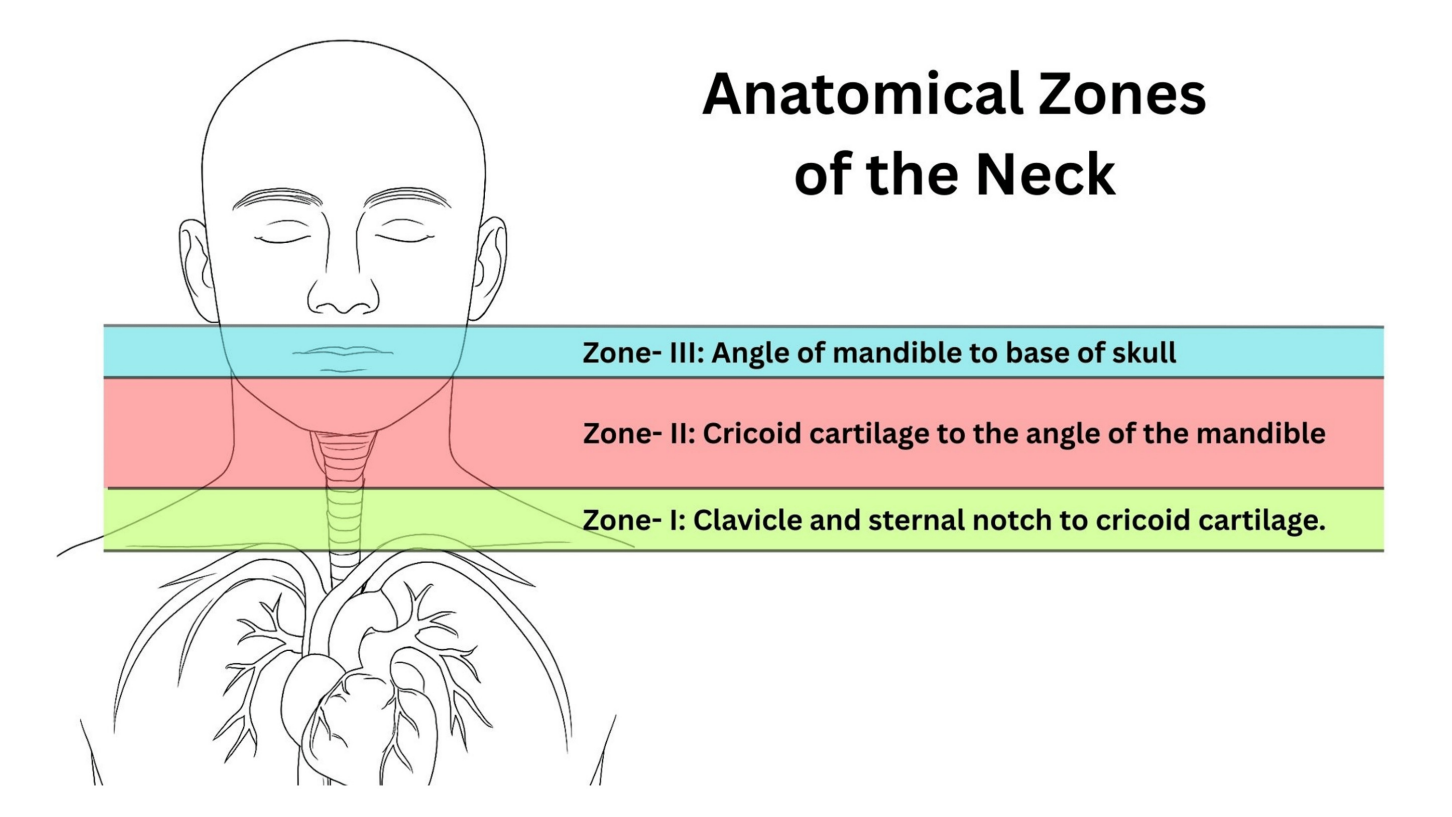
Anatomical zones of the neck

**Figure 2:**
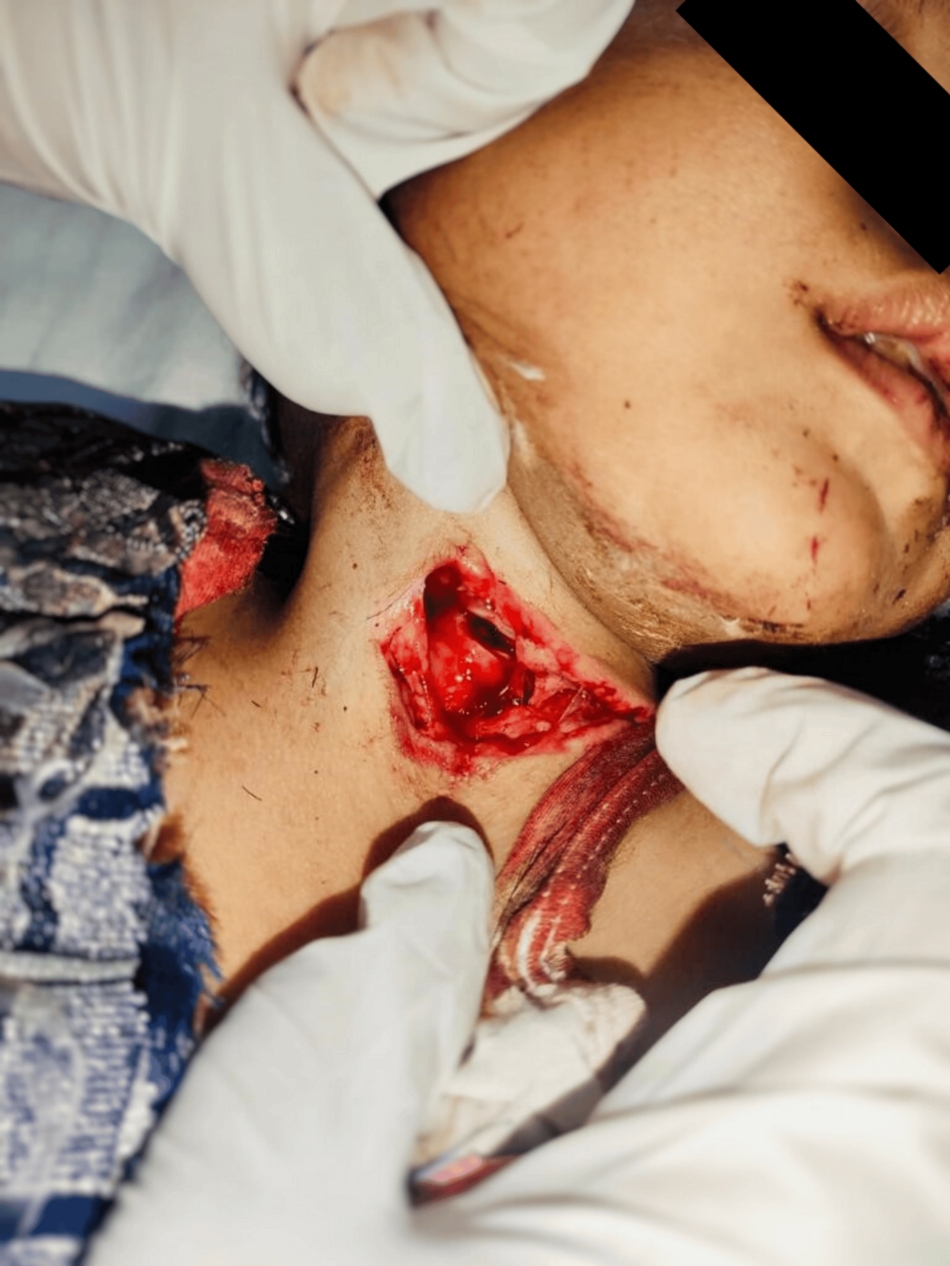
Anterior view of neck laceration with cricoid membrane tear

A four-year-old girl was brought to the trauma emergency room with a deep PNI in zone II (Figure 1). Examination revealed a 1 cm tear in the cricoid membrane (Figure 2). As the cause of the injury was unknown, a medical-legal case workup was initiated to investigate the circumstances and ensure appropriate documentation for forensic and legal purposes. She was bleeding from her neck, which made it difficult for her to breathe properly. During the examination, a laceration was found on the front of her neck, and a partial tear in the cricoid membrane was detected just below her thyroid gland. Air leaking from the injury was also noticed. On further examination, no injury to major nerves and blood vessels was identified, and the oesophagus was found to be intact. Her initial vital signs were recorded as body temperature of 37.2 °C, heart rate of 152 beats per minute, blood pressure of 92/70 mmHg, breathing rate of 26 breaths per minute, blood oxygen level of 98%, and Glasgow Coma Scale score of E4V2M6. In pediatric trauma, the Glasgow Coma Scale (GCS) is a standardized tool to assess consciousness in head injuries. It evaluates eye, verbal, and motor responses using age-appropriate criteria. Scores of 13-15 indicate mild, 9-12 moderate, and ≤8 severe injury, often requiring airway protection. The GCS is vital for early neurological assessment and intervention [9]. 

Upon admission, the patient underwent a primary survey (A, B, C, D, and E assessment) to assess and prioritise interventions. The airway was secured by intubating below the injury using video laryngoscopy. A dressing was applied to the laceration. Large-bore vascular lines were established, blood samples were obtained, and saline infusion was initiated. Computed tomography (CT) and X-ray confirmed a cricoid membrane tear without involvement of other vital neck structures. The patient received IV antibiotics (ceftriaxone and metronidazole) due to the risk of mediastinitis.

The patient’s laboratory results (Table 1) were within normal limits. On the same day, a skilled trauma surgeon performed a neck surgical intervention and successfully repaired the cricoid membrane without compromising any major neurovascular structures.

**Table 1:** Lab reports of the blood sample collected at the day of admission.

S.N.	Investigation	Patient value	Paediatric reference range
1	Haemoglobin	14 g/dL	11.5–14.5 g/dL
2	Haematocrit	39 %	34–40 %
3	Total leukocyte count	8.7 × 10⁹/L	5.0–14.5 × 10⁹/L
4	Platelet count	249 × 10⁹/L	150–450 × 10⁹/L
5	PT / INR	12.5 sec / 1.15	PT: 11–14 sec / INR: 0.8–1.2
6	Viral markers	Non-reactive	Non-reactive
7	Sodium	138 mmol/L	135–145 mmol/L
8	Potassium	3.6 mmol/L	3.5–5.0 mmol/L

Following surgery, the patient was transferred to the trauma critical care unit, where she received ventilatory support and one unit of packed red blood cells (pRBCs) (prophylactically due to prolonged operative time and increased intraoperative blood loss). On the second postoperative day, chest radiography and arterial blood gas (ABG) analysis were performed in accordance with the trauma center protocol, instituted due to a recent increase in hospital-acquired pneumonia and guided by the ICU antibiogram. Pediatric consultation highlighted a risk of chest infection, leading to escalation of antibiotics to intravenous meropenem. By the third day, the patient improved significantly, was extubated, remained stable, and was transferred to the trauma surgery ward.

Following extubation, a nasopharyngeal airway was placed to maintain airway patency. Regular oral suctioning and nebulization were performed every two-hour interval to ensure airway clearance. Oral sips of normal saline-soaked gauze were initiated, followed by gradual introduction of clear liquids such as oral rehydration solution (ORS) and coconut water. Over the subsequent two days, daily chest radiographs, ABG analysis, and vital sign monitoring were performed in accordance with ICU antibiogram protocol to exclude chest infection, given the recent rise in hospital-acquired pneumonia. 

The patient’s recovery was uneventful, with stable hemodynamics. The surgical wound demonstrated satisfactory healing, with regular dressing changes performed using sterile saline-soaked gauze to maintain cleanliness and promote tissue repair. She was able to eat and drink without difficulty, and there was no change in her voice. The patient was discharged on the eighth day after primary repair, with appropriate follow-up guidance. At the one-week follow-up, there were no signs of infection or other complications, chest radiography revealed no abnormalities, and the surgical wound continued to show satisfactory healing without significant scarring.

Informed consent was obtained from the patient's relatives, and hospital approval was obtained before presenting this case report.

## Discussion

The neck may be small, but it houses crucial structures from various body systems, including the cardiovascular, respiratory, neurologic, digestive, and musculoskeletal systems, making PNIs life-threatening and challenging. Sharp objects like knives and glass, as well as ballistic injuries, account for 5-10% of trauma cases [10]. Tracheal injuries are rare (less than 1% of all traumatic injuries and 14% of PNIs) but can be severe, with a 10% mortality rate often exacerbated by severe bleeding [11].

PNIs most commonly occur in Zone II (Figure 1) and are often complicated by potential damage to vascular, airway, or esophageal structures [12]. Clinical signs of tracheal injury include breathlessness, hoarseness, coughing, and air leakage through the wound. Ensuring immediate airway patency remains the primary priority. In our case, the patient was admitted to the trauma centre with a Zone II PNI. The cause of the injury was unknown, resulting in a 1-cm partial tear of the cricoid membrane. Despite the injury, the patient remained hemodynamically stable without significant vascular bleeding. Airway was stabilized using video laryngoscopy to intubate below the injury. A dressing was applied to the laceration, and intravenous saline infusion was initiated. No evidence of digestive tract injury was identified, although such involvement is reported in nearly 50% of patients with airway trauma [13].

**Figure 3:**
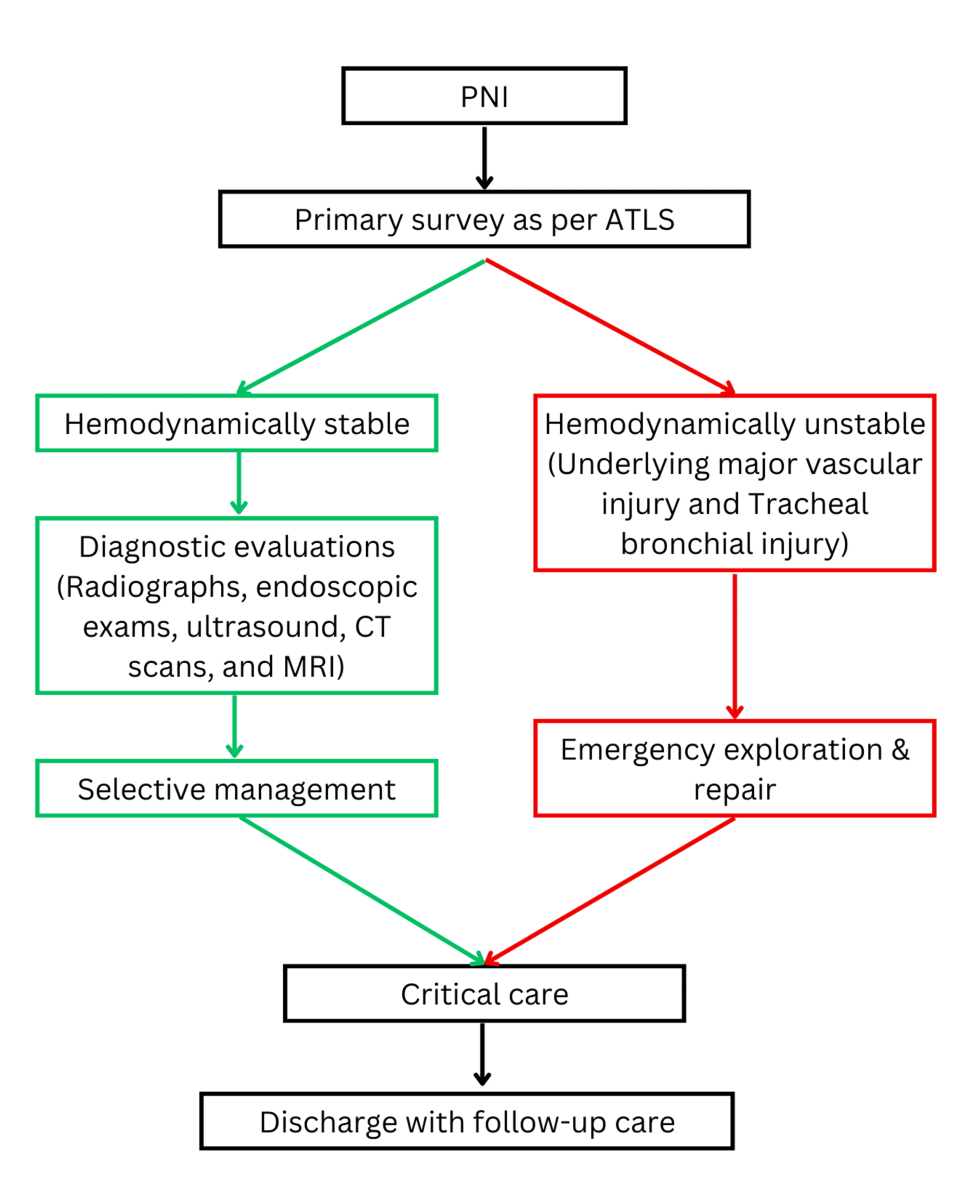
Proposed plan of management for penetrating neck injuries (PNIs).

Patients with neck trauma should first be stabilized according to ATLS protocols before diagnostic imaging (Figure 3). Radiographs of the neck and chest, along with bronchoscopy, help in determining the location, size, and depth of the injury. Common diagnostic tools include radiographs, endoscopy, ultrasound, CT, and MRI. In this case, CT and X-ray imaging confirmed a 1-cm tracheal tear in the cricoid membrane, with no injury to other vital neck structures. Prompt diagnosis and management are essential, as delayed recognition of tracheal rupture can lead to serious complications such as subcutaneous emphysema, pneumothorax, pneumomediastinum, and mediastinitis [14], which are associated with increased morbidity and mortality.

The patient’s physiological status and clinical findings during the primary survey (ABCDE assessment) guided our management. Contrary to the previous practice, where emergency surgery is seen as the standard for PNIs, conservative approaches are now increasingly employed [15]. While surgical exploration remains indicated for unstable patients presenting with expanding hematoma, air leakage, subcutaneous emphysema, hoarseness, or dysphonia, the primary survey (ABCDE assessment) combined with appropriate diagnostic modalities such as CT, ultrasound, and endoscopy can help avoid unnecessary surgical exploration in hemodynamically stable patients [16]. In this case, the patient was hemodynamically stable with no significant bleeding. On the same day, a trauma surgeon performed cricoid membrane repair and initiated antibiotic therapy to reduce the risk of mediastinitis. The patient was successfully weaned from the ventilator by the third postoperative day and, on the eighth day, was discharged with stable hemodynamics, no new complications, and appropriate follow-up guidance.

In conclusion, effective management of PNIs involves prompt assessment, tailored treatments, and careful follow-up .

## Conclusions

PNIs are rare yet critical emergencies that demand swift recognition and immediate intervention due to the complexity of the neck's vital structures. These injuries, often caused by sharp objects or projectiles, account for a significant portion of trauma cases and carry a considerable mortality risk, particularly when severe bleeding occurs. Promptly securing the airway and stabilizing circulation is paramount within the first hour, often referred to as the golden hour, to mitigate further complications. While the approach to treating these injuries has evolved from mandatory exploration to more selective management, ongoing debates surround the optimal strategies.

Our case report highlighted the intricacies of managing a PNI, specifically a cricoid membrane tear in a four-year-old patient. By promptly addressing the patient's stable hemodynamics, securing the airway through careful intubation, and conducting a successful surgical intervention and repair, we demonstrated the importance of tailored and comprehensive management. 
